# Co-occurrence of *Yersinia pestis* and a lytic bacteriophage in brain tissue of *Eothenomys miletus*

**DOI:** 10.1128/spectrum.03461-25

**Published:** 2026-09-30

**Authors:** Dongyue Lyu, Chao Su, Zhengliang Chai, Ennian Pu, Ziyi Bu, Jingdong Song, Shuai Qin, Ran Duan, Linxuan Yang, Zhuowei Li, Meng Xiao, Zhaokai He, Deming Tang, Peng Zhang, Lianxu Xia, Huaiqi Jing, Xin Wang, Zihou Gao

**Affiliations:** 1 National Institute for Communicable Disease Control and Prevention, Chinese Center for Disease Control and Prevention 12415, Beijing, China; 2 Yunnan Institute for Endemic Disease Control and Prevention 718973, Dali, Yunnan, China; 3 Anhui Medical University Clinic Medical College 654145, Hefei City, Anhui, China; 4 National Institute of Pathogen Biology, Chinese Academy of Medical Sciences, Peking Union Medical College 12501, Beijing, China; 5 Hangzhou Center for Disease Control and Prevention 117870, Hangzhou City, Zhejiang, China; 6 Dongcheng Center for Disease Control and Prevention, Public Health Emergency Management Innovation Center 607527 , Beijing, China; Creighton University, Omaha, Nebraska, USA

**Keywords:** *Yersinia pestis*, bacteriophage, natural plague focus, co-occurrence

## Abstract

We surveyed plague in the *Apodemus chevrieri–Eothenomys miletus* natural plague focus on the Yunnan Plateau, China. From 2023 to 2025, plague-positive *E. miletus* were detected in Gaomei, Xiaomachang, and Tangshang. In the brain tissue of an *E. miletus* carcass, both *Yersinia pestis* and a *Yersinia pestis*-specific bacteriophage (YN2024) were simultaneously isolated, and their simultaneous presence was observed via transmission electron microscopy (TEM). This observation was supported by previous analysis of a similar co-occurrence of *Y. pestis* and bacteriophage from the *Marmota himalayana* plague focus. After 72 h of *in vitro* liquid culture, TEM imaging revealed multiple morphological stages that were indicative of interaction, including intact bacteria, bacteria enveloped internally and externally with phages, lysed bacteria, and numerous released phages. These findings suggest that such co-occurrence may be a recurring phenomenon across different plague foci, as it has now been observed in two distinct ecosystems. Genomic analysis demonstrated that the phage YN2024 exhibited a high degree of homology with the phage AKS2021-55F0913 from the *M. himalayana* plague focus (average nucleotide identity, 96.94%). A comparison of growth curves indicated that the lytic capacity of phage AKS2021-55F0913 was stronger than that of phage YN2024.

**IMPORTANCE:**

Overall, our findings support the recognition of an ecologically connected *Apodemus chevrieri–Eothenomys miletus* natural plague focus and also provide a basis for understanding the co-occurrence of *Yersinia pestis* and its phage in natural hosts across multiple foci. The observed ecological linkage among plague foci previously attributed to different administrative regions highlights the importance of considering ecological connectivity in plague surveillance and control. In addition, these findings may contribute to a better understanding of host-phage interactions involving *Y. pestis* under natural conditions and may inform future phage-related studies in plague research.

## INTRODUCTION

Bacteriophages, viruses that specifically infect bacteria, have been widely documented in nature for over a century, and they exhibit unique life cycles and relatively specific host ranges (1). Research on bacteriophages has gained recent attention in the context of preventing and controlling bacterial infections, and there have been numerous explorations of the roles of bacteriophages in antibacterial immunity and alternative therapies. Specific bacteriophages of *Yersinia pestis* were discovered a century ago by d’Herelle (2, 3), who attempted to use them in treating four cases of bubonic plague via direct injection into the enlarged lymph nodes of patients, and certain therapeutic effects were reported (4, 5). However, alongside the swift advancement of antibiotic therapies (6, 7), research on bacteriophage therapy targeting *Y. pestis* declined, and there were no subsequent reliable reports of clinical application. Recent studies have revealed a symbiotic relationship between *Y. pestis* and a lytic phage (8, 9) that are present in the bone marrow of plague-infected deceased marmots. However, this relationship is extremely difficult to replicate under *in vitro* culture conditions. This phenomenon also poses limitations for using lytic *Y. pestis* phages to treat an acute infectious disease. To date, only one case has been observed where repeated cycles of lysis and regrowth occurred under *in vitro* culture conditions (8). During surveillance in the *Apodemus chevrieri–Eothenomys miletus* natural plague focus, we identified a co-occurrence between *Y. pestis* and a lytic *Y. pestis* phage in the brain tissue of *E. miletus* and conducted genomic analysis and further biological research.

## MATERIALS AND METHODS

### Sample sources

**Fig 1:**
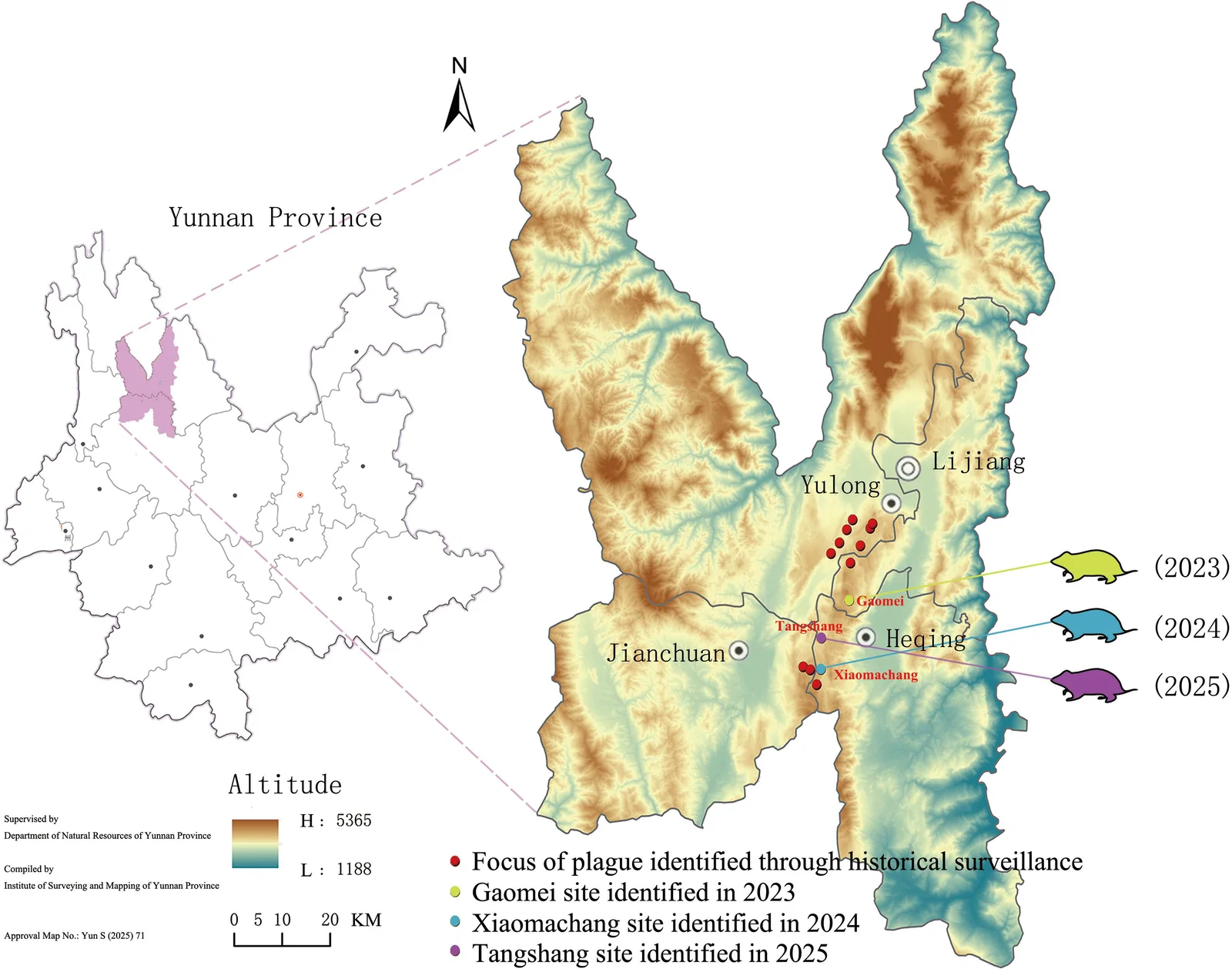
The *Apodemus chevrieri–Eothenomys miletus* plague focus on the Yunnan Plateau. *Y. pestis–*phage co-occurrence in brain tissue*.*

To gain a more comprehensive understanding of the characteristics of the *A. chevrieri–E. miletus* natural plague focus on the Yunnan Plateau, we conducted enhanced surveillance within this endemic area. Historically, this plague focus was divided into four distinct regions: Heqing County, Yulong County, Jianchuan County, and the Old Town of Lijiang (Fig. 1). Geographically, these sites are located along the southern edge of the Hengduan Mountains, with elevations ranging from 2,500 to 4,000 m. The local vegetation comprises subtropical broadleaf forests and subtropical pine forests, and the plateau has a monsoon climate that is characterized by distinct dry and wet seasons. The plague focus is distributed in the interconnected mountainous terrain, grasslands, and agricultural areas. Abundant vegetation and cultivated crops provide substantial food resources for rodent populations such as *E. miletus* and foster sustained population growth. From 2023 to 2025, three specialized, large-scale surveys employed board-trapping methods to capture wild rodents, from which tissue samples were collected for testing (10). The sampling strategy was designed according to the requirements of the National Plague Surveillance Protocol (2024 Revision) (11) for the *A. chevrieri–E. miletus* plague focus. Sampling sites were selected based on the following criteria: (i) fixed surveillance sites were established in areas with historical plague surveillance records indicating previous detection of *Y. pestis*; (ii) mobile surveillance was conducted by rotating and systematically covering administrative villages across the four administrative regions, with attention to accessibility for systematic surveillance. All carcasses encountered during systematic searching of the surveillance area were collected, regardless of their decomposition state, and subjected to bacteriological testing as required by the protocol. To minimize contamination risk, the samples were transported to the laboratory under sealed conditions. According to the biosecurity regulations, all samples must be collected under biosafety level 2 laboratory conditions, and personnel must don N95 respirators, biohazard protection suits, double-layered rubber gloves, and goggles. The intact skull was aseptically opened, and brain tissue was immediately collected under sterile conditions for subsequent bacterial isolation, nucleic acid extraction, and phage-related analyses.

### Detection and isolation of *Y. pestis* from live rodent tissue samples

Nucleic acids were extracted from live rodent tissue samples using a blood and tissue kit (69506, Qiagen, Germany). Polymerase chain reaction (PCR) targeting the *Y. pestis-*specific genes *pla* and *caf1* was performed to detect the presence of the pathogen (12). Tissue samples that tested positive for both *pla* and *caf1* genes were inoculated onto *Yersinia* selective medium and incubated at 28°C for 48 h to isolate strains (13).

### Detection and isolation of *Y. pestis* from rodent carcass tissue samples

The presence of the *Y. pestis* F1 antigen in tissue samples obtained from rodent carcasses was assessed using a reverse indirect hemagglutination assay (RIHA), and a titer of ≥1:100 indicated a positive result (14). Additionally, PCR was employed to detect the *pla* and *caf1* genes in these samples (12). Tissue specimens yielding positive results by either immunological assay or PCR were subsequently inoculated onto *Yersinia* selective medium and cultured at 28°C for 48 h to isolate strains (13).

### Isolation, proliferation, and electron microscopy examination of *Y. pestis*-specific phages

PCR screening for bacteriophages was conducted on tissue samples. Samples that tested positive for bacteriophage DNA were suspended in saline solution, centrifuged at 1,500 rpm for 3 min, and sequentially filtered through 0.45 and 0.22 µm filters to obtain a bacteriophage culture solution, which was used for co-isolation with *Y. pestis* EV76 to proliferate the bacteriophage (8).

After proliferation, the titer of phage YN2024 was determined to be 8 × 10^8^ plaque-forming units (PFU)/mL. The phage samples were stained with potassium phosphotungstate (pH 7.2) and observed under a transmission electron microscope. Images were captured and analyzed, and taxonomic classification was performed in accordance with the bacteriophage classification framework established by Ackermann and Berthiaume, as well as guidelines from the International Committee on Taxonomy of Viruses (12).

### Genome sequencing and assembly

Genomic DNA from *Y. pestis* was extracted using a Wizard Genomic DNA Purification Kit (Promega, USA), and a 10 K SMRT Bell library was constructed using a SMRT Bell template kit (v2.0). After sequencing by PacBio Sequel, assembly was performed using Canu (v2.0), and bacteriophage sequences in the genome were predicted by phiSpy (v2.3).

Bacteriophage genomic DNA was extracted using a sodium dodecyl sulfate lysis method and ultrasonically broken down to approximately 350 bp using a Covaris ultrasonic disruptor. The library was constructed using a NEB Ultra DNA library preparation kit and sequenced on an Illumina platform. Based on the De Bruijn algorithm, the phage genome was assembled with SPAdes and SOAPdenovo, and the open reading frame was predicted using Prokka (9).

### Determination of the growth curve

The lytic ability of bacteriophages was tested in *Y. pestis* EV76. Strain EV76 was inoculated into Brucella broth, and bacterial suspensions were adjusted to McFarland turbidity (MCF) standards of 0.5 and 1.0. Each MCF bacterial suspension was divided into three groups (A, B, and C), with six parallel samples in each group. In Group A, 300 μL of the bacterial suspension was mixed with 30 μL of Brucella broth. In Group B, 300 μL of the bacterial suspension was mixed with 30 μL of 2.4 × 10^7^ PFU of phage YN2024. In Group C, 300 μL of the bacterial suspension was mixed with 30 μL of 2.4 × 10^7^ PFU of phage AKS2021-55F0913. The multiplicity of infection (MOI) is defined as the ratio of phage plaque-forming units to bacterial colony-forming units (CFUs) added to the culture mixture. At McFarland 1.0 (approximately 2.4 × 10^7^ CFU/mL), the addition of 2.4 × 10^7^ PFU of phage yielded an MOI of 1.0; at McFarland 0.5 (approximately 1.2 × 10^7^ CFU/mL), the MOI was 0.5. The blank control was Brucella broth. Honeycomb plates containing the A, B, and C mixtures were incubated in a growth curve analyzer for 24 h, and the optical density at 600 nm (OD_600_) was recorded every 30 min. The data represent the average values of three independent experiments. Statistical analysis was performed using analysis of variance, and a *P*-value <0.05 was considered statistically significant.

## RESULTS

### Plague prevalence in the *A. chevrieri–E. miletus* plague focus

From 2023 to 2025, a total of 722 live rodents were captured and 23 carcasses were collected during surveillance of the *A. chevrieri–E. miletus* plague focus. The rodent species included *A. chevrieri*, *E. miletus*, *Rattus flavipectus*, *Apodemus draco*, and *Crocidura attenuata*, with *A. chevrieri* and *E. miletus* being the dominant species (Table 1).

**TABLE 1:** Species in the *Apodemus chevrieri–Eothenomys miletus* plague focus on the Yunnan Plateau

Year	Capture type	Species	Number	Number of positive cases of plague
2023	Live	*Apodemus chevrieri*	56	0
		*Rattus flavipectus*	1	0
	Carcass^*a*^	*Apodemus chevrieri*	3	0
		*Eothenomys miletus*	9	2
2024	Live	*Apodemus chevrieri*	209	0
		*Eothenomys miletus*	75	0
		*Rattus flavipectus*	5	0
		*Rattus nitidus*	1	0
		*Apodemus draco*	2	0
		*Crocidura attenuata*	5	0
	Carcass	*Apodemus chevrieri*	1	0
		*Eothenomys miletus*	2	1
2025	Live	*Apodemus chevrieri*	229	0
		*Eothenomys miletus*	129	0
		*Rattus flavipectus*	2	0
		*Apodemus latronum*	2	0
		*Micromys minutus‌*	2	0
		*Crocidura attenuata*	4	0
	Carcass	*Eothenomys miletus*	3	1
		*Rattus flavipectus*	3	0
		*Rattus norvegicus*	1	0

^*a*^ One rat was found among the carcasses, but its species could not be identified.

In 2023, 57 live mice and 13 carcasses were collected, with a flea detection rate of 1.43% (1/70). From one *E. miletus* carcass, *Y. pestis* was isolated from the organs and embryos. Another carcass tested positive for *Y. pestis* via PCR, but bacterial isolation was unsuccessful. In 2024, 297 live mice and 3 carcasses were collected, with a flea detection rate of 29.3% (88/300). *Y. pestis* (YN24HQzi2) and *Y. pestis*-specific phage YN2024 were simultaneously isolated from the brain tissue of an *E. miletus* carcass (YN202411-HQzi2). In 2025, 368 live mice and 7 carcasses were collected (of which 4 were found in the ditch of a residential area), with a flea detection rate of 33.6% (126/375). One *E. miletus* carcass bone marrow RIHA was positive, and PCR and sequencing of the *pla* and *caf1* genes from brain tissue confirmed the presence of *Y. pestis*.

Throughout 2023–2025, plague-positive animals were identified in Gaomei, Xiaomachang, and Tangshang, thereby confirming these locations as active plague foci. The identification of these sites as new plague foci effectively addressed previous surveillance gaps. These locations share the same continuous landscape, host species, and vectors, suggesting that they are ecologically connected and may constitute a larger natural plague focus. However, formal proof of epidemiological continuity would require additional genetic evidence, such as whole-genome sequencing of isolates from different subregions (Fig. 1).

### *Y. pestis–*phage co-occurrence in brain tissue

**Fig 2:**
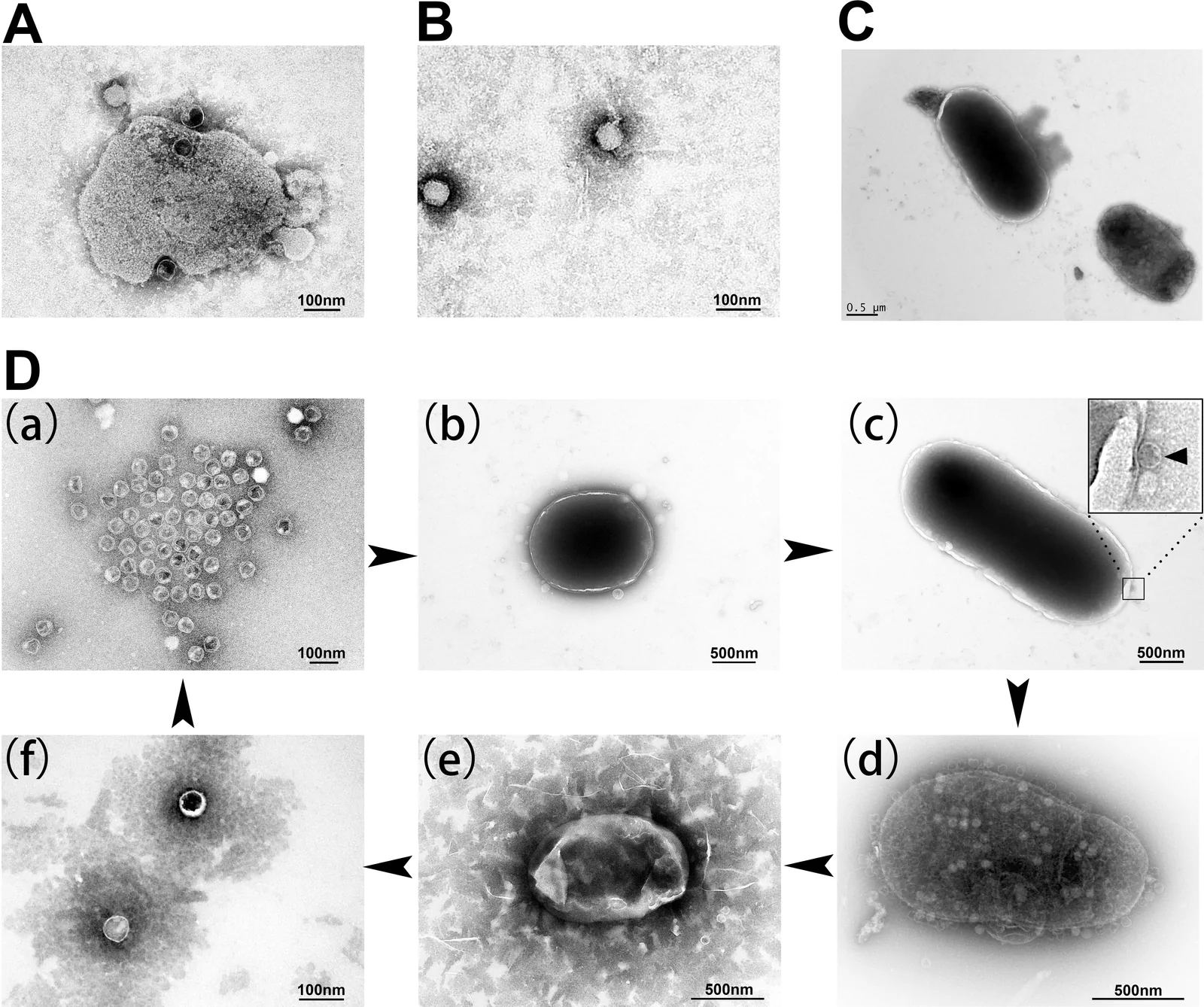
Electron micrographs of *Y. pestis* and its specific phage. (**A**) Phage YN2024 and *Y. pestis* YN24HQzi2 isolated from the brain tissue of *E. miletus* from the *A. chevrieri–E. miletus* plague focus. (**B**) Characteristic electron micrograph of phage YN2024 in the *Podoviridae* family. (**C**) *Y. pestis* from *M. himalayana* in its natural plague focus, showing bacteria in different states (e.g., infected vs uninfected/resistant) at the same time point. (**D**) Electron micrographs showing coexisting *Y. pestis* and phages at different stages of infection or resistance: (a) clusters of phage, (b) *Y. pestis* uninfected or potentially resistant to phage infection, (c) phages adsorbed to the surface of infected *Y. pestis* and injecting their nucleic acid, (d) phage synthesis and assembly within *Y. pestis*, (e) lysed dead *Y. pestis*, and (f) phages released following lysis of *Y. pestis*.

An *E. miletus* carcass (YN202411-HQzi2) discovered in 2024 was severely decomposed upon collection, which prevented gender identification or procurement of visceral tissues. However, the skull remained intact, and the relatively preserved brain tissue was immersed in physiological saline for nucleic acid extraction. This sample tested positive for the *Y. pestis*-specific genes *pla* and *caf1*. Inoculation of the brain tissue subsequently led to the simultaneous isolation of *Y. pestis* and a *Y. pestis*-specific phage (as observed via transmission electron microscopy [TEM], Fig. 2A).

The isolated phage exhibited a tadpole-like morphology typical of the *Podoviridae* family and was characterized by an isometric hexagonal head and a short, non-contractile, conical tail (Fig. 2B).

We previously observed a similar process within the *M. himalayana* natural plague focus on the Qinghai-Tibet Plateau (8). Electron micrographs of bacteria from *M. himalayana* showed the simultaneous presence of *Y. pestis* unaffected by phages and *Y. pestis* at various stages of infection (Fig. 2C). Further *in vitro* culture observations revealed these bacteria to be at different stages of the phage infection cycle, which includes adsorption, injection, intracellular replication, lysis, and release, or potentially in a defensive/resistant state (Fig. 2D), thus illustrating a classic evolutionary arms race.

### Gene sequencing and genomic comparison of *Y. pestis* and phage from brain tissue: the genome of *Y. pestis* YN24HQzi2

The genome of *Y. pestis* YN24HQzi2 (GenBank accession JBRANZ000000000), isolated from an *E. miletus* carcass (YN202411-HQzi2), was 4,647,252 bp in size, with a GC content of 47.63%. It carried pCD, pPCP, and pMT1 plasmids. Prophage sequences and five CRISPR arrays were predicted within the genome, indicating the presence of CRISPR-associated anti-phage defense systems. However, none of the spacer sequences within the CRISPR arrays matched the sequence of the isolated phage YN2024.

### Genomic composition, homology, and evolutionary analysis of phage YN2024

The genome of phage YN2024 (GenBank accession PX390146) was 37,748 bp in size, with a GC content of 47.41%. Annotation against the SwissProt database identified 40 predicted genes, 37 of which had known functions, while 3 were hypothetical proteins. Functional module analysis revealed genes related to gene regulation, DNA replication, restriction-modification, genome packaging, morphogenesis, and host lysis, which were consistent with the conserved features of the *Podoviridae* family. No bacterial virulence genes were detected. Gene Ontology classification indicated that the genes of phage YN2024 were primarily involved in molecular functions (e.g., DNA binding and hydrolase activity), biological processes (e.g., viral replication), and cellular components (e.g., phage capsid).

**Fig 3:**
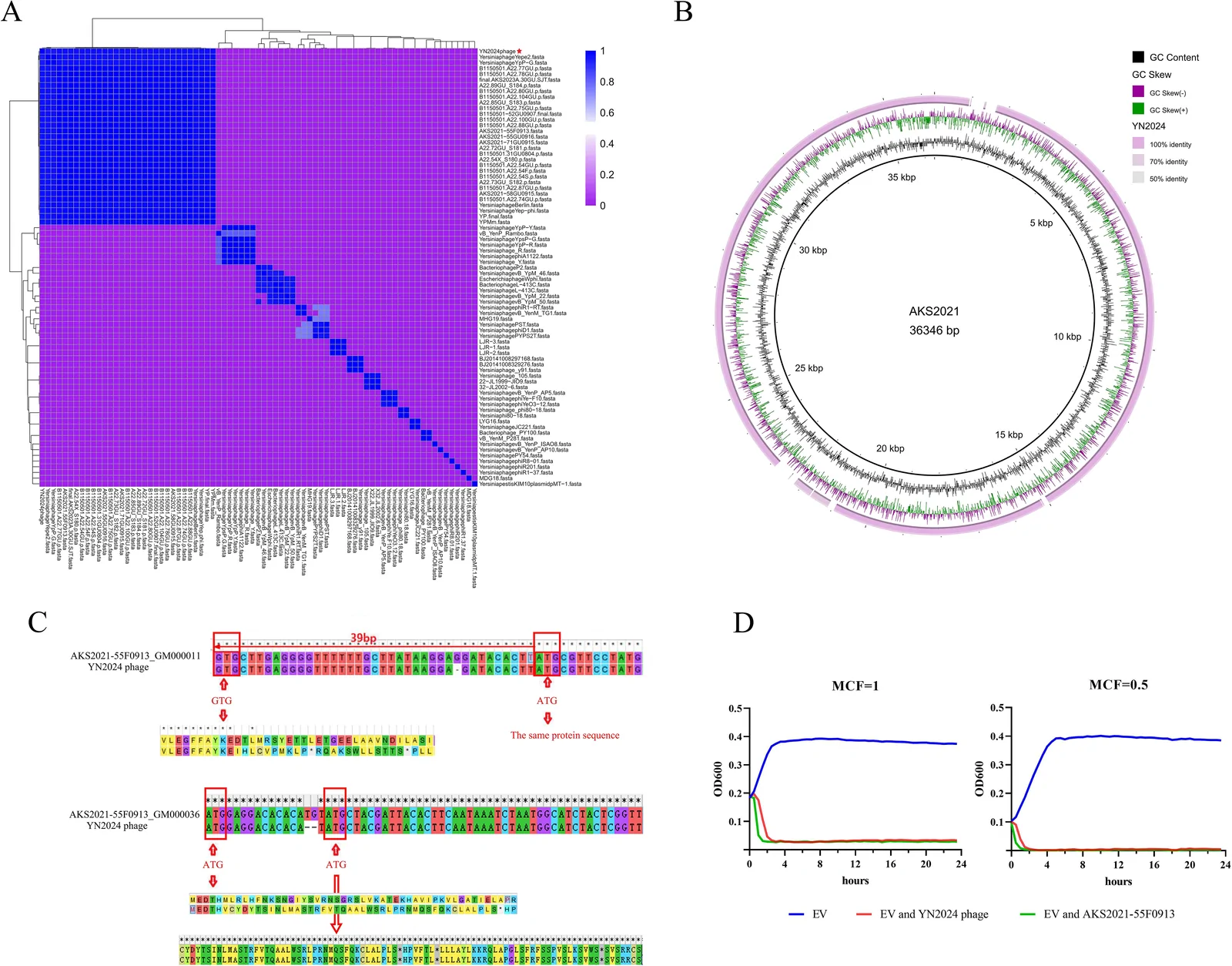
Phage homology analysis and growth curves. (**A**) ANI matrix of various phages. Phage YN2024 isolated in this study is marked with a ★. (**B**) Homology analysis between phage YN2024 and phage AKS2021-55F0913. (**C**) Frameshift mutations identified in the genome of phage YN2024. (**D**) Growth curves of phage YN2024 and phage AKS2021-55F0913.

The genomic sequence of phage YN2024 showed high homology with previously isolated lytic *Y. pestis* phages, with average nucleotide identity (ANI) values ranging from 96.09% to 96.94%. The highest homology was observed with phage AKS2021-55F0913. Phylogenetic analysis demonstrated that phage YN2024 clustered closely with various known lytic *Y. pestis* phages (e.g., Yep-phi, Berlin, YpP-G, and Yepe2), which all share greater than 94% homology. In contrast, phage YN2024 showed no significant homology with temperate *Yersinia* phages, which formed a distinct clade (Fig. 3A). The phage YN2024 sequence was not integrated into the host bacterium’s chromosome. These findings indicate that phage YN2024 is a lytic bacteriophage.

### Comparative genomic analysis of phage YN2024

A whole-genome alignment was performed using the phage AKS2021-55F0913, which shares the highest homology with phage YN2024, as the reference sequence. The analysis revealed that phage YN2024 lacked four coding sequences (CDSs) present in the reference sequence. These missing CDSs encode a RecBCD nuclease inhibitor, a type VII endonuclease, and a tail fiber protein (Fig. 3B). Furthermore, frameshift mutations were identified in two CDSs of the phage YN2024 sequence compared to the reference sequence: a single-base deletion and a two-base insertion. These mutations are located in another tail fiber protein-coding gene (affecting the 10th amino acid) and in a BC10 family protein gene (affecting the 6th amino acid). Analysis of the predicted protein sequences suggests that translation likely initiates from nearby start codons and produces proteins whose lengths fully encompass the functional domains. Consequently, these proteins are expected to retain functionality, including the key functional protein gp11 (15), which affects phage proliferation (Fig. 3C). Conversely, the reference phage AKS2021-55F0913 lacks six CDSs found in the phage YN2024 sequence, including genes encoding a DNA polymerase and an HNH endonuclease.

### Growth curves of phage YN2024 and phage AKS2021-55F0913

Growth curves for phage YN2024 and phage AKS2021-55F0913 were generated based on OD_600_ measurements recorded every 30 min (Fig. 3D). The OD_600_ of the *Y. pestis* EV76 control culture (without phage) increased initially and then reached a plateau.

For cultures infected with phage YN2024 at a multiplicity of infection (MOI) of 1.0, OD_600_ began to decrease after 0.6 h, and complete lysis required 2.1 h. At an MOI of 0.5, OD_600_ started decreasing approximately 0.3 h post-infection, and complete lysis was achieved within 2.1 h.

In comparison, the OD_600_ decrease was faster in cultures infected with phage AKS2021-55F0913. At an MOI of 1.0, OD_600_ began dropping after 0.4 h. At an MOI of 0.5, OD_600_ decreased shortly after incubation began, and complete lysis required only 1 h. These results indicate that the lytic capability of phage AKS2021-55F0913 is stronger than that of phage YN2024. This difference was significant at the *P* < 0.05 level (Table S1).

## DISCUSSION

Since their identification, natural plague foci in Yunnan Province have sustained uninterrupted enzootic transmission along with sporadic human cases (10). Plague is an ecologically determined zoonosis; distinct foci express distinct epidemiological signatures. Two canonical focus types occur in Yunnan: a wild-rodent *A. chevrieri–E. miletus* focus and a commensal-rat focus centered on *R. flavipectus*. Although animal-to-animal transmission persists in both types, the risk of human infection is substantially higher in the commensal-rat focus. Commensal rats live in close proximity to human dwellings and are predominantly infested with *Xenopsylla cheopis*, which readily develops proventricular blockage, thus enhancing transmission. In contrast, *E. miletus* is chiefly parasitized by *Ctenophthalmus* fleas, and plague rarely spills over to humans (16).

Shifts in habitat structure and host distributions will likely render routine surveillance insufficient in delineating the true extent of plague in wild-rodent foci. To address this, three targeted investigations were conducted from 2023 to 2025. The 2023 survey partially overlapped with local rodent control campaigns, which limited the sample yield. Across 3 years, 722 live rodents and 23 carcasses were collected. All live specimens tested negative for *Y. pestis*-specific genes, and four carcasses tested positive via PCR. An additional four carcasses collected from drainage ditches near residential areas were PCR-negative and were suspected rodenticide deaths. Intensified sampling closed prior boundary gaps among four subregions of the wild-rodent focus (17). This finding reflects the true low prevalence characteristic of the *A. chevrieri-E. miletus* plague focus. Epidemiological data from Yunnan Province demonstrate that this focus type is characterized by prolonged quiescent intervals and very low seropositivity in live animals, with *Y. pestis* detection rates considerably lower than those observed in the highly active *M. himalayana* focus on the Qinghai-Tibet Plateau (10). Concordant ecological structure, vegetation, and the shared assemblage of principal hosts and vectors suggest that these zones are ecologically connected and may constitute a larger natural focus—a reasonable epidemiological inference based on standard field surveillance criteria. However, this inference does not constitute genetic proof of true epidemiological continuity. Whole-genome sequencing of *Y. pestis* isolates from the different positive locations would be required to determine whether they belong to the same transmission network. Therefore, while the surveillance has successfully closed previous gaps, the inference of a single contiguous focus remains tentative pending genetic evidence.

Phage therapy for plague remains unrealized a century after d’Herelle’s early attempts (4). While our understanding of phage biology has advanced and includes genomics, receptor mapping, and host-phage interaction (6), work on plague-specific phages has lagged. *Y. pestis* phage genomes are highly conserved, and virion morphology is comparatively uniform (8, 9, 18), which is consistent with our prior observations (9). Due to their narrow lytic spectra and high host specificity (19, 20), the principal global utility of *Y. pestis* phage genomes remains a diagnostic identification tool rather than a therapeutic tool. Our field and laboratory data offer an ecological–molecular rationale for the pervasive *Y. pestis*–phage co-occurrence across foci and hosts that is difficult to reconstitute *ex vivo*, yet persists in naturally decomposing animal remains (8, 9).

The carcass from which *Y. pestis* and phage YN2024 were co-isolated showed evident external decomposition, although the brain tissue remained relatively well preserved, likely due to the protective effect of an intact skull. The postmortem interval could not be accurately determined under field conditions. Consequently, the possibility of postmortem microbial redistribution or environmental contamination cannot be fully excluded. However, several factors suggest that such interference was likely limited. First, the intact cranial structure may have provided a physically enclosed microenvironment that reduced exposure to external microbial invasion and environmental fluctuations. Second, all sampling procedures were conducted under aseptic and biosafety-controlled conditions, minimizing the risk of exogenous contamination during specimen collection and processing. Carcass decomposition is a highly dynamic process characterized by progressive changes in temperature, pH, oxygen availability, and the release of host-derived enzymes and microbial metabolites, all of which can influence microbial viability and community structure. As previously summarized by Jończyk et al. (21), the stability and persistence of bacteriophages are strongly influenced by external physical and chemical conditions, although phage tolerance to adverse environments varies considerably among different viral taxa. In this context, bacterial autolysis during decomposition may also contribute to the release of intracellular phages, thereby potentially facilitating their recovery from postmortem tissues. Taken together, while decomposition introduces inherent uncertainty, the successful co-occurrence of *Y. pestis* and phage YN2024 is consistent with the known environmental resilience and persistence of bacteriophages under variable and often unfavorable conditions.

Through TEM, this study revealed that phages and bacteria were engaged in a continuous dynamic process of infection, resistance, mutation, and reinfection. Furthermore, previous animal studies have indicated that *Y. pestis*-specific phages fail to effectively eliminate bacteria residing within macrophages, leading to a rebound in bacterial load during the late stage of infection (22, 23). This complex relationship in a natural host is likely a key reason for the lack of substantial breakthroughs in plague phage therapy research over the past century. The co-occurrence of phages and bacteria in natural hosts, together with bacterial anti-phage defenses, suggests that the *in vivo* efficacy of phage therapy may be more complex than predicted by *in vitro* experiments. This observation may partially explain the historical difficulties in achieving consistent therapeutic success. This hypothesis requires direct experimental validation. Particularly in highly contagious, rapidly progressing, and highly fatal clinical forms of plague, such as pneumonic plague, this relationship severely limits the application potential of single-phage therapy strategies (24).

Genomic analysis further supports this view. *Y. pestis* develops resistance mutations and employs multiple mechanisms to defend against phage infection. Different phages typically rely on recognizing specific surface proteins on a host bacterium to complete adsorption (25, 26), and mutations in or loss of bacterial surface protein receptors can lead to reduced or even completely ineffective phage adsorption. The *psaA* gene carried by strain YN24HQzi2 encodes a pilus adhesin. Previous studies suggest that its expression product may non-specifically hinder plague phages from accessing key receptors by masking them (27). In addition, various anti-phage defense systems in *Y. pestis* (28, 29), such as CRISPR-Cas and Retron systems (30–33), enhance bacterial resistance by recording information from previous phage infections and mounting corresponding immune responses, thereby forming a complex defense network. Analysis of the *Y. pestis* YN24HQzi2 genome confirmed its potential for CRISPR-Cas-mediated anti-phage defense. A lack of sequence matches to phage YN2024 may have occurred because the CRISPR spacers in *Y. pestis* YN24HQzi2 evolved in response to other phages, or because phage YN2024 is a recent variant generated through genetic mutation or horizontal gene transfer. This possibility also reflects an ongoing arms race and dynamic equilibrium between bacteria and phages in nature (34).

Bacteriophages can also adapt to host bacterial changes via genomic variation (such as receptor-binding proteins and anti-defense genes) (25, 35). The deletion of CDSs from reference sequences (such as those encoding the fiber protein in the genome of phage YN2024) may affect infection efficiency and host range. In addition, anti-defense system genes such as the *Acr* family gene were not detected in the genome of phage YN2024. The Acr protein corresponds to resistance to the CRISPR-Cas defense system (36), which can help phages evade immune clearance. Biological curve analysis showed that the lytic ability of phage YN2024 was not as strong as that of phage AKS2021-55F0913 isolated from the *M. himalayana* plague focus (Fig. 3D). This difference may be closely related to the virulence background of the host strain. The same result was found in previous studies of the *M. himalayana* plague focus (9), and as the area with the highest plague intensity and strongest strain toxicity in China, highly virulent *Y. pestis* and bacteriophages compete with each other in the *M. himalayana* plague focus; thus, it may be easier to screen phage populations with stronger lytic capabilities through adaptive mutations. The underlying mechanism remains to be explored.

Although EV76 enables standardized amplification and cross-comparison of different phages under uniform conditions, it may not fully recapitulate the phage-host dynamics that occur in the natural host background. The single amplification step using EV76 may have introduced a selection bias, and the characterized phage YN2024 may represent only a subset of the original phage population. Moreover, the lytic activity of the phage was assessed using EV76 rather than the original host isolate YN24HQzi2. Furthermore, no *in vivo* experiments were performed to directly demonstrate how the observed phage-bacterium co-occurrence affects plague pathogenesis, transmission, or bacterial fitness within the host. Future studies employing metagenomic sequencing and other culture-independent approaches, together with host range and lytic kinetics tests on YN24HQzi2 and a broader panel of local *Y. pestis* isolates, will help to more accurately resolve the native phage community structure in natural hosts and minimize the artificial selection introduced by culture-based methods.

In this study, the identification of a *Y. pestis*–phage co-occurrence in the brain tissue of *E. miletus* suggests that the two organisms may reach a balance in such immune barrier areas as the central nervous system. This discovery provides a new perspective on the persistence of *Y. pestis* in the host. Similarly, in cold, high-altitude regions such as the Qinghai–Tibet Plateau, animal carcasses (such as marmots) decay slowly and can mummify after a few months. *Y. pestis* and bacteriophages (8) can still be isolated from the bone marrow of carcasses, which confirms the prevalence of co-occurrence of *Y. pestis* and the possibility of symbioses in natural epidemic foci.

## Data Availability

The genome sequences of *Yersinia pestis* YN24HQzi2 and bacteriophage YN2024 have been deposited in GenBank under accession numbers JBRANZ000000000 and PX390146, respectively. All other data generated or analyzed during this study are included in the main text or the supplemental material.

## SUPPLEMENTAL MATERIAL

Table S1 spectrum.03461-25-s0001.docx YN2024 phage and AKS2021-55F0913 at two MCFs with different time points.

ASM does not own the copyrights to Supplemental Material that may be linked to, or accessed through, an article. The authors have granted ASM a non-exclusive, world-wide license to publish the Supplemental Material files. Please contact the corresponding author directly for reuse.
