# Supplementary material for: Co-occurrence of Yersinia pestis and a lytic bacteriophage in brain tissue of Eothenomys miletus
Source: Microbiol Spectr. 2026 Sep 30;14(11):e03461-25. doi: 10.1128/spectrum.03461-25 (PMC13729188; doi:10.1128/spectrum.03461-25)
Supplement: Supplementary file 1 [file spectrum.03461-25-s0001.docx]

| Table S1: Statistical analysis between YN2024 phage and AKS2021-55F0913 at two MCFs with different time points | | | | | | |
| --- | --- | --- | --- | --- | --- | --- |
| MCF | Time (h) | F (Sig.) | group | Sig.(Pairwise comparison) | 95% CI | |
|  |  |  |  |  | Lower | Upper |
| 1.0 | 0.6 | 897.4 (0.00*) | A vs B | 0.0077* | 0.007261 | 0.03774 |
|  |  |  | A vs C | <0.0001* | 0.04332 | 0.07361 |
|  |  |  | B vs C | <0.0001* | 0.02693 | 0.04501 |
|  | 0.4 |  | A vs B | 0.3989 | -0.007628 | 0.02116 |
|  |  |  | A vs C | 0.0214* | 0.003122 | 0.03188 |
|  |  |  | B vs C | 0.0096* | 0.002898 | 0.01857 |
| 0.5 | 0.3 | 797.4 (0.00*) | A vs B | 0.0066* | 0.005595 | 0.02834 |
|  |  |  | A vs C | <0.0001* | 0.04557 | 0.06763 |
|  |  |  | B vs C | <0.0001* | 0.03239 | 0.04688 |
|  | 0.1 |  | A vs B | 0.3529 | -0.006378 | 0.02058 |
|  |  |  | A vs C | 0.0018* | 0.01091 | 0.03615 |
|  |  |  | B vs C | 0.0044* | 0.00598 | 0.02689 |
| *. The mean difference is significant at the 0.05 level. Group A: culture solution without phages infected Group B: culture solution infected with bacteriophage YN2024 phage Group C: culture solution infected with bacteriophage AKS2021-55F0913 | | | | | | |
